# Preserving Subjective Wellbeing in the Face of Psychopathology: Buffering Effects of Personal Strengths and Resources

**DOI:** 10.1371/journal.pone.0150867

**Published:** 2016-03-10

**Authors:** Elisabeth H. Bos, Evelien Snippe, Peter de Jonge, Bertus F. Jeronimus

**Affiliations:** University of Groningen, University Medical Center Groningen, Interdisciplinary Center Psychopathology and Emotion regulation, Groningen, The Netherlands; Technion—Israel Institute of Technology, ISRAEL

## Abstract

**Background:**

Many studies on resilience have shown that people can succeed in preserving mental health after a traumatic event. Less is known about whether and how people can preserve subjective wellbeing in the presence of psychopathology. We examined to what extent psychopathology can co-exist with acceptable levels of subjective wellbeing and which personal strengths and resources moderate the association between psychopathology and wellbeing.

**Methods:**

Questionnaire data on wellbeing (Manchester Short Assessment of Quality of Life/Happiness Index), psychological symptoms (Depression Anxiety Stress Scales), and personal strengths and resources (humor, Humor Style questionnaire; empathy, Empathy Quotient questionnaire; social company; religion; daytime activities, Living situation questionnaire) were collected in a population-based internet study (HowNutsAreTheDutch; N = 12,503). Data of the subset of participants who completed the above questionnaires (n = 2411) were used for the present study. Regression analyses were performed to predict wellbeing from symptoms, resources, and their interactions.

**Results:**

Satisfactory levels of wellbeing (happiness score 6 or higher) were found in a substantial proportion of the participants with psychological symptoms (58% and 30% of those with moderate and severe symptom levels, respectively). The association between symptoms and wellbeing was large and negative (-0.67, P < .001), but less so in persons with high levels of self-defeating humor and in those with a partner and/or pet. Several of the personal strengths and resources had a positive main effect on wellbeing, especially self-enhancing humor, having a partner, and daytime activities.

**Conclusions:**

Cultivating personal strengths and resources, like humor, social/animal company, and daily occupations, may help people preserve acceptable levels of wellbeing despite the presence of symptoms of depression, anxiety, and stress.

## Introduction

In 1946, the World Health Organization defined health as “a state of complete physical, social, and emotional wellbeing, and not merely the absence of disease or infirmity” [1]. It is by now conventional wisdom that the absence of somatic or psychological symptoms is not a sufficient condition for high levels of subjective wellbeing [2]. Similarly, the presence of symptoms does not necessarily imply low wellbeing. For example, people suffering from ill-health or disease may still feel happy and satisfied with their life [3–5], in keeping with the Stoic argument that each person has the capacity to be happy, no matter how daunting and painful the circumstances of life might be. Individuals with somatic or mental health complaints are more than the sum of their symptoms, and also have intact faculties, character strengths, and positive experiences [6,7]. These may counteract or buffer against the negative impact of symptoms. Thus, while the absence of symptoms is not a guarantee for wellbeing, the presence of symptoms does not preclude it.

This latter observation fits in with the large body of literature on resilience after trauma. Traumatic experiences are known to increase the risk of psychopathology, but surprisingly many people do not develop mental health problems after trauma (50–90%) [8,9]. Several factors underlying resilience have been proposed, of which autonomy, a sense of mastery or internal locus of control, self-esteem, humor, purpose (including religion and spirituality), and social support are among the most frequently reported [10–12].

Most research on resilience has focused on preserving mental health after a traumatic event. Less research has been devoted to preserving a sense of subjective wellbeing in the presence of psychopathology [2]. People may deal differently with symptoms because they differ in their personal strengths and resources, and therefore may retain different levels of subjective wellbeing despite of these symptoms. Although in some areas of psychotherapy there has been a shift from an exclusive focus on symptoms to the cultivation of strengths and positive outcomes [6,13,14], it remains unclear to what extent people with psychological symptoms can preserve a sense of wellbeing and which factors contribute to such “wellness within illness” [5].

The aim of the present study is (a) to examine to what extent psychopathology co-exists with acceptable levels of subjective wellbeing and (b) which personal strengths and resources moderate the association between psychopathology and subjective wellbeing. To do so, subjective wellbeing and a broad range of symptoms in the domains of depression, anxiety and stress were examined in a sample from the general Dutch population. The literature distinguishes three components of subjective wellbeing: affect (momentary moods and emotions), domain satisfactions, and global judgements of life satisfaction or happiness [15,16]. In this study, we focused on global judgements of life satisfaction and happiness because i) we were not interested in the short-term fluctuations of momentary affect, and ii) we did not want to contaminate our wellbeing measure with specific domain satisfactions like work, social relations, or personal autonomy, because these factors may actually *contribute* to global life satisfaction, and the degree to which may differ between individuals. This is in line with strategies of the United Nations [17] and offices of national statistics [18,19], who use these global measures of life satisfaction and happiness in their annual surveys. Regarding personal strengths and resources, we focused on factors that were not inherently confounded with wellbeing, because one pitfall of research on resilience is that predictors and outcome overlap or reflect the same underlying psychological construct [20]. In other words, we selected factors that may *promote* wellbeing but are not part of wellbeing. As mentioned above, confining our wellbeing measure to global judgments of life satisfaction and happiness was one way to prevent this overlap. Further, we selected measures of personal strengths and resources that did not show content overlap with this wellbeing measure. A number of established resilience factors were selected from the existing literature, including humor, religious belonging, and having a partner. These factors were supplemented with some less established factors, including having a pet, daytime activities, and empathy. We expected a buffering effect of these psychosocial resources, that is, we expected that the negative effects of psychological symptoms on subjective wellbeing is attenuated in individuals with higher levels of these resources. Further, in a multivariable analysis we examined which of these factors are most important in preserving subjective wellbeing in the presence of psychopathology.

## Methods

### Participants and Procedure

Participants from the general population of the Netherlands were recruited by means of a crowdsourcing procedure, in a call to participate in research on mental health as a dimensional and dynamic phenomenon, by means of radio broadcasts, television, local podium discussions, newspapers, and magazines. In this project, called HowNutsAreTheDutch (in Dutch: HoeGekIsNL), individuals were invited to visit the website www.HoeGekIs.nl (also www.HowNutsAreTheDutch.com) and to assess themselves on their mental health in a cross-sectional internet study. Participants could fill out questionnaires on several domains of mental health, including sociodemography, wellbeing, psychopathology, and various psychosocial strengths and resources. These questionnaires were presented on the website in “modules”, that is, a set of questionnaires covering a specific domain. The order in which the modules could be completed was partly fixed. All modules were visible from the start, but initially only some of them were activated. The first mandatory module was the “Start” module, assessing participants’ sociodemographic profile. Subsequently, participants got access to three key modules: 1) Affect/mood; 2) Wellbeing; and 3) Living situation, which could be completed in any order. After the Affect/Mood and Wellbeing modules had been completed all other modules became available and could be completed in any order. Most of the questionnaires were existing and well-validated questionnaires, except the Living situation questionnaire. Details on the HowNutsAreTheDutch project are provided elsewhere [21].

The present study is based upon the 12,503 individuals who participated in the cross-sectional study between December 19^th^ 2013 (launching date of the internet platform) and December 13th 2014 (end of first-year wave). Inclusion criteria were adulthood (18^+^) and informed consent on use of the data for scientific research. No formal written or oral consent was obtained because the data were analyzed anonymously. The Medical Ethical Committee of the University Medical Center Groningen approved the study procedures and declared the study was excepted from review by the Medical Research Involving Human Subjects Act (in Dutch: WMO) because it was a non-randomized open study targeting anonymous volunteers in the general population (number M13.147422). For the present study we selected those participants who filled out all the questionnaires relevant to this study and who filled out the questionnaires on psychopathology and wellbeing within one and the same week. This resulted in a final sample of 2411 participants.

### Measures

#### Subjective Wellbeing

The wellbeing module of the HowNutsAreTheDutch study included the Manchester Short Assessment of Quality of Life (MANSA) [22], the Happiness Index (HI) [23], the Ryff scales of psychological wellbeing (Ryff) [15], and the Social Production Function Instrument for the Level of wellbeing (SPF-IL) [24]. As explained in the introduction, in this study, we focused on global judgments of life satisfaction and not on specific domain satisfactions like work, social relations, or personal autonomy [15]. Therefore, we selected those items of the above-mentioned questionnaires that exclusively assess global judgments of life satisfaction. One of the items we selected was the first item of the Manchester Short Assessment of Quality of Life [22], which rates global life satisfaction with the question “How satisfied are you with your life as a whole at this moment?”. Participants answered this question on a 7-point scale ranging from 1 = “Couldn’t be worse”, via 4 = “Mixed (satisfied and unsatisfied)”, to 7 = “Couldn’t be better”. Global life satisfaction is considered as a prominent indicator of subjective wellbeing in the literature [7,15]. The second item we selected was happiness as assessed with the Happiness index, which measures people’s happiness with a single item [23]. Participants rated the question “Do you feel happy in general?” on an 11-point scale (0–10). Previous work showed this single item to be reliable and valid in community surveys, and to show good concurrent validity (with other happiness/wellbeing/life satisfaction questionnaires) and divergent validity [23,25]. Happiness is often used interchangeably with subjective wellbeing and many researchers argue that happiness may be typically what people have in mind when they refer to wellbeing [26–28]. We calculated a composite score for subjective wellbeing from these two items by standardizing and averaging them. We did so for statistical reasons, that is, to enhance the reliability of the wellbeing measure. Cronbach’s α for this measure was 0.87.

#### Psychopathology

Psychopathology was assessed in the Affect/Mood module of the HowNutsAreTheDutch study, which included the Depression Anxiety Stress Scales (DASS) [29,30], the Quick Inventory of Depressive Symptomatology (QIDS) [31], and the PANAS [32]. In this study we focused on the DASS, because this instrument covers three common domains of psychological symptoms, viz. depression, anxiety and stress, and is known to be sensitive to subthreshold symptoms [29,30]. The DASS assesses depression, anxiety, and stress over the past week. The questionnaire consists of 42 self-report items (14 items per scale) that each tap into a negative psychological or psychosomatic symptom. The DASS subscales show close similarity to the tripartite model [33]. The Depression scale assesses dysphoria, hopelessness, devaluation of life, self-deprecation, lack of interest/involvement, anhedonia, and inertia. The Anxiety scale assesses autonomic arousal, skeletal musculature effects, situational anxiety, and subjective experience of anxious affect. The Stress scale assesses difficulty relaxing, nervous arousal, and being easily upset/agitated, irritable/over-reactive and impatient. Questions include “I felt sad and depressed”, “I was aware of dryness of my mouth”, and “I found it hard to wind down”. Each item is rated on a 4-point Likert scale ranging from 0 = “Did not apply to me at all” to 3 = “Applied to me very much, or most of the time”. For each subscale, a sum score was calculated. Internal consistency was sufficient in our study, viz., for depression, anxiety, and stress Cronbach’s α was 0.96, 0.89, and 0.92, respectively. The subscales were summed to derive a total symptom score (DASS Total). This score was used in the analyses. We used this total score of the DASS because were interested in global levels of psychopathology rather than in specific symptom domains. Moreover, the three symptom scales of the DASS show high intercorrelations, and there are theoretical reasons as well as empirical evidence that especially the depression and anxiety subscales are not independent constructs at all [34].

#### Humor styles

Humor was assessed with the Humor Style Questionnaire (HSQ), which distinguishes affiliative, self-enhancing, aggressive, and self-defeating humor [35] The HSQ contains 32 items (8 for each subscale) which are rated on a Likert scale ranging from 1 = “totally disagree” to 7 = “totally agree”. The subscales refer to potentially beneficial and detrimental ways in which people tend to make use of humor in everyday life. Affiliative humor refers to the use of humor to amuse others and facilitate relationships (Cronbach’s alpha = .89). Self-enhancing humor refers to use of humor to cope with stress and maintain a humorous outlook during times of difficulty (α = .87). Aggressive humor refers to usage of sarcastic, manipulative, put-down, or disparaging humor (α = .70). Self-defeating humor refers to excessive self-disparagement, ingratiation, or defensive denial (α = .82). We examined the differential effects of these different humor styles, because some of these may act as resilience factors, while others may be detrimental.

#### Empathy

Empathy was assessed with the 40-item Empathy Quotient (EQ) questionnaire, which captures both shared emotions and cognitive empathy or theory of mind [36]. The EQ is intended to measure how easily participants pick up on other people’s feelings and how strongly they are affected by them (e.g., “I can easily tell if someone else wants to enter a conversation”). Each item is rated on a 4-point Likert scale ranging from 0 = “strongly agree” to 3 = “strongly disagree”. The EQ scale had a Cronbach’s alpha of 0.89.

#### Other Factors

We assessed whether or not participants had a romantic partner, a pet, a religious belonging, and daytime activities (including paid work, unpaid work, school, household activities, and retirement) using the living situation questionnaire of the HowNutsAreTheDutch study [21]; S1 Text.

### Analytic Approach

Descriptive statistics were used to examine the distribution of subjective wellbeing scores among participants with different symptom severities. Ordinary least squares regression analyses were performed to examine the association between symptomatology and subjective wellbeing and the moderating effect of personal resources. Wellbeing scores were modeled as a function of symptoms (DASS Total score), personal resources, and the interaction between symptoms and resources. This was done for each resource separately. Next, a multivariable regression analysis was performed in which all resources and all interactions between symptoms and resources were included, to identify the most important resources. All models were adjusted for sex, age, and education, because it is well-known that these factors are related to psychopathology as well as wellbeing, and presumably also to some of the personal strengths and resources. Non-significant effects were trimmed from this multivariable model one by one. All continuous variables were standardized into z-scores by subtracting the mean and dividing by the standard deviation, so the regression coefficients can be interpreted as standardized effect sizes (beta’s). We classified betas as small if between 0.10 and 0.20, moderate between 0.20 and 0.30, and large if above 0.30, based on the effect sizes commonly found in social psychology [37,38]. Categorical variables (Having a partner, Having a pet, Religious belonging, Daytime activities) were coded 0 = no, 1 = yes. For all models, bootstrapped 95% confidence intervals based on 1000 replications are presented. Analyses were performed using SPSS Statistics 23 (IBM, SPSS Inc., Chicago, IL). A p-value of 0.05 was used for statistical significance.

## Results

### Sample Characteristics

Of the 2411 participants included in the study, 1557 were female (65%). Mean age was 49.5 years (SD = 13.7, range 18–81). The majority of the participants was highly educated (81% high, 15% middle, 4% low). 73% of the participants had a partner, 46% had a pet, 25% belonged to a religious denomination, and 93% reported to have daytime activities. Average symptom levels were low, but the scores spanned the entire range of DASS subscales; DASS Depression scores ranged from 0 to 42 (mean = 6.0, SD = 7.3); DASS Anxiety scores ranged from 0 to 40 (mean = 3.1, SD = 4.3); DASS Stress scores ranged from 0 to 42 (mean = 7.7, SD = 6.6); and the DASS Total scores ranged from 0 to 120 (mean = 16.7, SD = 16.0). According to the DASS manual [24], the following cut-offs can be used for the Depression subscale (normal/mild<14, moderate 14–20, severe/extremely severe≥21), the Anxiety subscale (normal/mild<10, moderate 10–14, severe/extremely severe≥15), and the Stress subscale (normal/mild<19, moderate 19–25, severe/extremely severe≥26). In our sample, 2066 participants (85%) had normal/mild symptom levels, whereas 207 participants (9%) had moderate symptom levels and 138 participants (6%) had severe or extremely severe symptom levels on one or more of the subscales. Note that we use these cut-offs purely for illustrative purposes, as DASS scores are meant to reflect a continuous underlying dimension [39].

### Subjective Wellbeing in Participants with Different Symptom Severity

**Fig 1:**
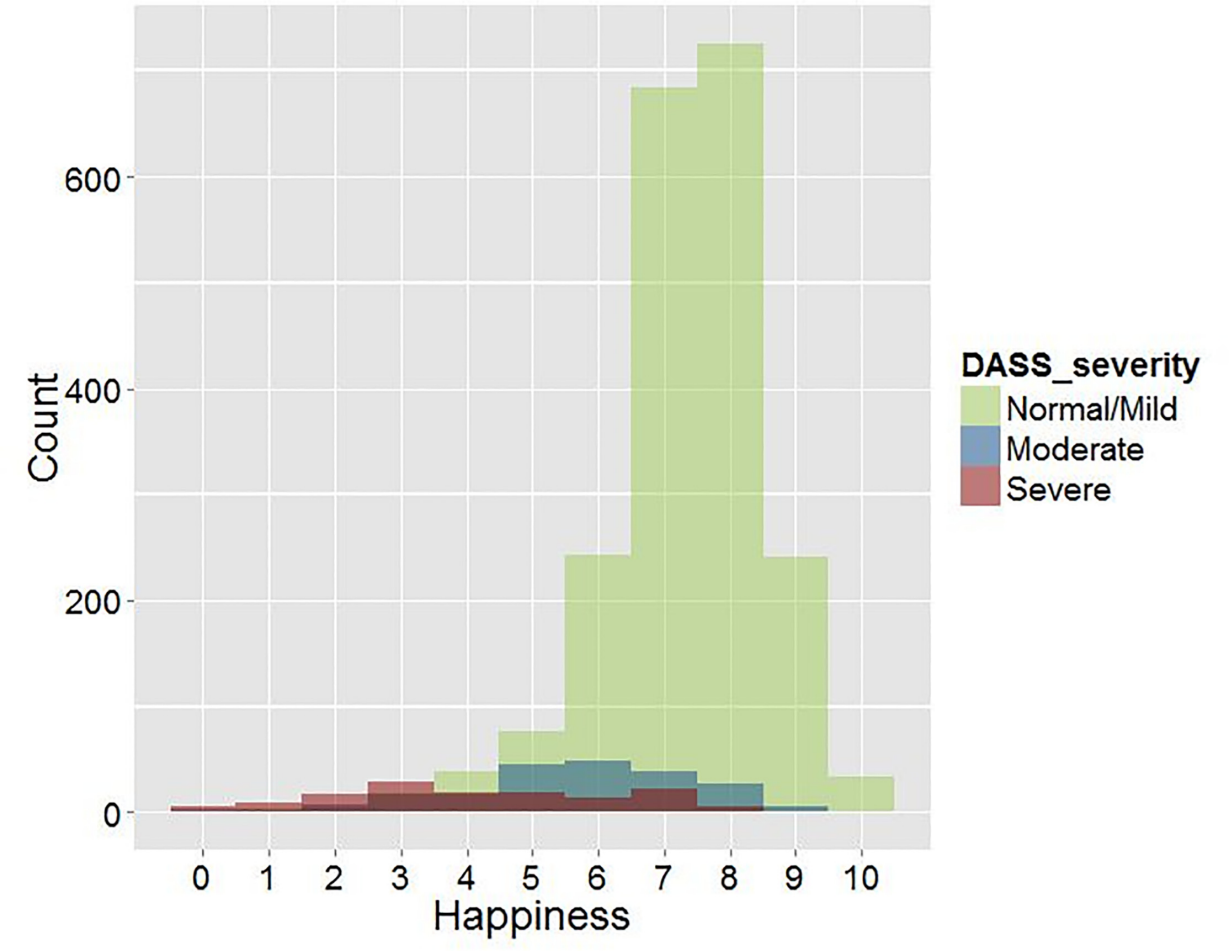
Distribution of happiness ratings in three symptom severity subgroups (N = 2411). Normal/Mild subgroup: mean (sd) = 7.3 (1.2), range 1–10; Moderate subgroup: mean (sd) = 5.7 (1.8), range 0–9; Severe subgroup: mean (sd) = 4.2 (2.2), range 0–9.

To compare happiness scores for participants with different symptom levels, we compared participants with normal/mild, moderate, and severe/extremely severe symptom levels on one or more of the subscales of the DASS. The results are presented in Fig 1. The Happiness Index is presented in the figure because we think that a happiness score is more easy to interpret than our composite wellbeing score. Nevertheless, life satisfaction scores and the composite wellbeing scores showed similar distributions.

The figure shows that most participants with low symptom scores report high happiness scores: 93% (n = 1927) of these participants rate their happiness as satisfactory (6 or higher). In the moderate symptoms subgroup, 58% (n = 119) of the participants rate their happiness at 6 or higher. In the subgroup with severe symptom levels, 30% (n = 42) of the participants rate their happiness at 6 or higher. The range in happiness scores was equal in all three subgroups (viz. 9 units). The standard deviation was substantially higher in the moderate and severe symptoms subgroups compared to the normal/mild subgroup (sd = 1.2 vs. 1.8 vs. 2.2; Levene statistic: 94.3 (df = 2408), p < .001).

### Moderating Effects of Personal Resources in the Association between Symptoms and Wellbeing

Without the resource variables in the model, the association between symptoms and wellbeing was large and negative (beta = -0.69, 95% bootstrapped CI -0.73 to -0.65, p < .001; results not shown in Table). Table 1 shows the results of the univariable regression analyses on the moderating effects of personal resources in the association between psychological symptoms and wellbeing. Three personal resources moderated the negative association between symptoms and wellbeing, viz. self-defeating humor, partner status, and having a pet. As expected, these moderating effects were positive, implying that the negative effect of symptoms on wellbeing was attenuated in persons with higher levels of self-defeating humor, with a partner, and/or with a pet. Thus, these resources seem to buffer against the negative effects of symptoms on wellbeing. The size of the moderating effects was small.

**Table 1:** Univariable regression analyses predicting wellbeing from symptoms, personal resources, and the interaction between symptoms and resources (n = 2411).

		95% bootstrapped CI		
	Beta	Lower	Upper	p
*Humor*				
Symptoms	**-0.661**	**-0.706**	**-0.617**	**< .001**
Affiliative humor	**0.120**	**0.089**	**0.150**	**< .001**
Symptoms*Affiliative	0.012	-0.027	0.052	.545
Symptoms	**-0.623**	**-0.671**	**-0.583**	**< .001**
Self-enhancing humor	**0.184**	**0.153**	**0.216**	**< .001**
Symptoms*Self-enhancing	0.005	-0.029	0.041	.743
Symptoms	**-0.687**	**-0.730**	**-0.645**	**< .001**
Aggressive humor	0.018	-0.015	0.049	.255
Symptoms*Aggressive	0.003	-0.035	0.042	.882
Symptoms	**-0.708**	**-0.757**	**-0.659**	**< .001**
Self-defeating humor	**-0.036**	**-0.068**	**-0.003**	**.034**
Symptoms*Self-defeating	**0.074**	**0.046**	**0.099**	**< .001**
*Social company*				
Symptoms	**-0.733**	**-0.792**	**-0.671**	**< .001**
Having a partner	**0.333**	**0.270**	**0.402**	**< .001**
Symptoms*Partner	**0.084**	**0.010**	**0.160**	**.030**
Symptoms	**-0.727**	**-0.788**	**-0.667**	**< .001**
Having a pet	0.013	-0.048	0.073	.631
Symptoms*Pet	**0.079**	**0.005**	**0.156**	**.036**
*Other resources*				
Symptoms	**-0.677**	**-0.719**	**-0.638**	**< .001**
Empathy	**0.097**	**0.068**	**0.130**	**< .001**
Symptoms*Empathy	0.027	-0.005	0.062	.120
Symptoms	**-0.676**	**-0.728**	**-0.627**	**< .001**
Religion	-0.023	-0.086	0.037	.463
Symptoms*Religion	-0.047	-0.129	0.039	.271
Symptoms	**-0.587**	**-0.701**	**-0.498**	**< .001**
Daytime activities	**0.350**	**0.214**	**0.493**	**< .001**
Symptoms*Daytime activities	-0.099	-0.201	0.017	.081

Note. Dependent variable: Wellbeing. All continuous variables are z-transformed, thus estimated coefficients equal standardized B values (Beta). Categorical variables 0 = no, 1 = yes. Adjusted for sex, age, and education. In bold: significant effects. CI = Confidence Interval.

The main effects of the personal resources on subjective wellbeing were significant for affiliative humor, self-enhancing humor, partner status, empathy, and daytime activities, such that higher scores on these resources were related to higher levels of wellbeing. The main effect of self-defeating humor was significant but had a negative sign, which implies that for people with average symptom levels, more self-defeating humor was related to lower levels of wellbeing (the main effects of the continuous variables apply to persons with average symptom levels in these analyses, since continuous variables were standardized). The size of the main effects ranged from small to large.

Table 2 shows the results of the multivariable regression analyses predicting wellbeing from symptoms, resources, and the interaction between symptoms and resources. This model supported the moderating effect of self-defeating humor and partner status in the association between symptoms and wellbeing. The moderating effect of having a pet became nonsignificant in this multivariable model, and was therefore removed from the model. The significant main effects of self-enhancing humor, self-defeating humor, partner status, empathy, and daytime activities persisted. Consistent with the univariate analyses, these main effects were positive except for self-defeating humor. The largest effect sizes were observed for partner status (beta = 0.33) and daytime activities (beta = 0.24).

**Table 2:** Multivariable regression analysis predicting wellbeing from symptoms, personal resources, and the interaction between symptoms and resources (n = 2411).

		95% bootstrapped CI		
	Beta	Lower	Upper	p
Symptoms	-0.665	-0.724	-0.606	< .001
Self-enhancing humor	0.191	0.157	0.223	< .001
Self-defeating humor	-0.069	-0.101	-0.038	< .001
Having a partner	0.333	0.269	0.390	< .001
Empathy	0.042	0.012	0.070	.011
Daytime activities	0.241	0.123	0.374	< .001
Symptoms*Self-defeating humor	0.066	0.039	0.094	< .001
Symptoms*Partner	0.091	0.023	0.159	.008

Note. Multivariable regression analyses; dependent variable: Wellbeing. All continuous variables are z-transformed, such that estimated coefficients are standardized B values (Beta). Having a partner 0 = no, 1 = yes. Adjusted for sex, age, and education. Non-significant effects have been removed from this model one by one. CI = Confidence Interval.

## Discussion

The current study provides evidence for the idea that acceptable levels of wellbeing can co-exist with significant psychopathology. Although higher levels of anxiety, depression, and stress predicted lower levels of wellbeing, a considerable proportion of the participants with moderate to severe symptom levels rated their happiness as satisfactory (58% and 30% of those with moderate and severe symptom levels, respectively). One reason for this finding may be that these participants can buffer against—or compensate for—the negative effect of symptoms by means of their personal strengths and resources. This argument is supported by our finding that the negative effect of symptoms on wellbeing is attenuated in individuals with higher levels of self-defeating humor, with a partner, and/or with a pet. Hence, these resources seem to have a protective effect on the detrimental effect of symptoms on wellbeing. Moreover, several personal resources showed a positive main effect on wellbeing, especially self-enhancing humor, having a partner, daytime activities, and empathy. This implies that these resources are advantageous regardless of the level of symptoms.

The observation that the presence of psychological symptoms does not rule out satisfactory levels of subjective wellbeing is consonant with studies by Bergsma et al. [40] and Palmer et al. [5], in which a substantial number of people with mental disorders showed moderate to high levels of happiness. Thus, some people can retain acceptable levels of subjective wellbeing despite of their symptoms. Such differences in resilience against symptomatology are not well accounted for in our psychiatric phenotypes, which are defined exclusively in terms of symptoms [41]. This may be one reason why research into the etiology of psychiatric disorders—based on these phenotypes—is often inconsistent, and why observed effect sizes are generally small [42,43].

The factor with the highest effect size in the multivariable analysis was having a partner, which was positively associated with wellbeing (main effect), especially in the presence of symptoms (interaction effect). This is consonant with the literature on resilience after trauma showing the stress-buffering effects of intimate social relationships [10,12]. Of course, it is not so easy to translate this finding to a concrete clinical intervention: getting a partner is not easy, especially not for people with mental health problems. Interestingly, our results suggest that the company of a pet may be a viable alternative. Although pets do not seem to increase wellbeing in people with average symptom levels, they do seem to mitigate the negative impact of symptoms at higher levels of psychopathology, in keeping with previous studies that showed that pets can increase positive affect among individuals suffering from depression [44] and can serve as important sources of social support [45]. Some general practitioners prescribe a dog for people with (subsyndromal) levels of depression and loneliness [46], with positive effects. A dog or another pet may not only provide social company, it may also increase opportunities for positive affect [47], enhance activity levels, and address mental faculties involved in care and empathy [48], which in turn may enhance subjective wellbeing [49,50].

We showed differential effects of different humor styles in the present study. As far as we know, this is the first study in which different humor styles are investigated with regard to their distress-buffering effects. Our analyses showed that self-enhancing humor was the humor style with the largest positive effect on wellbeing. Self-defeating humor was negatively related to wellbeing, but the moderating effect of this humor style on the association between symptoms and wellbeing was positive. This suggests that self-defeating humor is unfavorable in individuals with average symptom levels, but favorable in people with high symptom levels. The negative main effect found for self-defeating humor is consistent with findings by Martin et al. [35], who also found negative associations between this humor style and several measures of mood and wellbeing. These authors have suggested that aggressive and self-defeating humor are deleterious forms of humor. Our findings suggest that at higher symptom levels a little bit of self-irony may not be so bad at all. Humor may contribute to effective coping, and may be a way to exercise some control [51].

We did not find significant main or interaction effects for religious belonging. Religion or spirituality has been reported to be a resilience factor in studies on trauma by a number of authors [10,52]. Our negative finding may be explained by the fact that we measured whether people belonged to a religious denomination, which is not necessarily the same as having strong religious or spiritual beliefs and practices.

Daytime activities were positively associated with wellbeing in our study, in line with some previous observations [47,53]. This result supports the intuition that having a daily occupation is beneficial; it may provide meaning, distraction, day structure, social relations, and self-esteem [47,53]. Yet, we did not find a moderating effect of daytime activities. This may imply that daytime activities are beneficial regardless of the level of symptoms, but it may also reflect a lack of power; only 7% of our sample did not have daytime activities. It should also be noted that we cannot rule out reverse causality, viz. people may not be able to work *because* they have low levels of subjective wellbeing and high levels of symptoms. This is a limitation of our design.

The effects that were significant in this study were small to large in size compared to those generally observed in the psychological literature [38]. Their combined effect may especially be large, because the observed effects were additive: our multivariable analysis showed that the main effects of self-enhancing and self-defeating humor, having a partner, empathy, and daytime activities were independent of one another. The same was true for the moderating effects of having a partner and self-defeating humor. Moreover, some effects may accumulate over time via feedback loops with other traits and environmental factors [54]. Targeting multiple domains of personal strengths and resources may therefore be a fruitful strategy; the total effect of these strengths and resources largely outweighs the negative effect of symptoms.

Our study has implications for psychiatric research as well as clinical diagnostics and interventions. The results suggest that subtyping in psychiatric research should not be confined to identifying people with different symptomatology [55,56], but should also consider differences between people in wellbeing and personal strengths and resources. The results also substantiate calls for a more positive focus in clinical diagnosis and intervention [6,21,57]. For example, the World Psychiatric Association charged a workgroup for the development of a more comprehensive model of classification and diagnosis [58], including positive aspects of health and personal and social resources [57]. Furthermore, treatment and prevention strategies for psychiatric disorders may benefit from a shift from an exclusive focus on symptom reduction to strategies aimed at fostering strengths and resources. A number of studies have already shown some evidence for the effectiveness of interventions aimed at cultivating positive mental faculties and social resources [59–62]. Interestingly, these interventions do not need to be very encompassing. Relatively simple strategies, like “spend more time socializing”, “count your blessings”, and “perform one kind act a day” have shown to enhance feelings of wellbeing [25,47,50,63]. Such interventions are relatively easy to implement in daily life, and have no costs and no side effects.

Strengths of our study include the relatively large sample size and the inclusion of possible resilience factors that had not been studied before. A limitation of our study is the cross-sectional design, which prevents drawing conclusions about the direction of the associations. Another limitation is that our sample may not be representative of the general population, given the voluntary nature of the study (self-selection), and the relatively high educational levels and high proportion of females in our sample. Furthermore, the selection of personal resource variables was inevitably incomplete and to some extent arbitrary. One problem, however, with many constructs used in the resilience literature, such as mastery, self-acceptance, and optimism, is the risk of circularity or content overlap with wellbeing: so-called resilience factors may tap into the same underlying construct as wellbeing measures [20]. Another risk is that resilience factors overlap with symptom measures. For example, items on optimism may measure the same as items of depression questionnaires measuring pessimism about the future. For this reason, we selected variables that may be assumed to promote wellbeing but are not inherent to wellbeing or psychopathology. Finally, our wellbeing measure was confined to global judgments of life satisfaction and happiness, while the mood/affect component of wellbeing may vary from day to day [15,64]. The HowNutsAreTheDutch project also contains a diary study, which may be used in future studies to investigate which factors influence these daily fluctuations in feelings of wellbeing [21].

To conclude, this study shows that it is possible to maintain acceptable levels of wellbeing in the presence of psychopathology, and provides evidence that this may be explained by the compensatory and buffering effects of personal strengths and resources. Differences between individuals in such resources may be another source of heterogeneity in psychiatric research and practice. Cultivating these positive factors in therapy may help to supplement the traditional “fix-what’s-wrong” approach by a “build-what’s-strong” approach [6].

## Supporting Information

S1 Text Living situation questionnaire. (DOC)

## References

[1] World Health Organization. Constitution of the World-Health-Organization. Public Health Rep. 1946;61: 1268–1277. 20995760

[2] Keyes CLM. Promoting and protecting mental health as flourishing: A complementary strategy for improving national mental health. Am Psychol. 2007;62: 95–108. 17324035 10.1037/0003-066X.62.2.95

[3] Perkins R. What constitutes success? Br J Psychiat. 2001;179: 9–10. 10.1192/bjp.179.1.9 11435261

[4] Mezzich JE. Positive health: Conceptual place, dimensions and implications. Psychopathology. 2005;38: 177–179. 16145269 10.1159/000086086

[5] Palmer BW, Martin AS, Depp CA, Glorioso DK, Jeste DV. Wellness within illness: Happiness in schizophrenia. Schizophr Res. 2014;159: 151–156. 10.1016/j.schres.2014.07.027 25153363 PMC4928639

[6] Duckworth AL, Steen TA, Seligman MEP. Positive psychology in clinical practice. Annu Rev Clin Psychol. 2005;1: 629–651. 17716102 10.1146/annurev.clinpsy.1.102803.144154

[7] Sheldon KM, Kashdan TB, Steger MF, editors. Designing Positive Psychology: Taking Stock and Moving Forward. New York: Oxford University Press; 2011.

[8] Bonanno GA, Westphal M, Mancini AD. Resilience to loss and potential trauma. Annu Rev Clin Psychol. 2011;7: 511–535. 10.1146/annurev-clinpsy-032210-104526 21091190

[9] Masten AS. Ordinary magic—Resilience processes in development. Am Psychol. 2001;56: 227–238. 11315249 10.1037//0003-066x.56.3.227

[10] Southwick SM, Vythilingam M, Charney DS. The psychobiology of depression and resilience to stress: implications for prevention and treatment. Annu Rev Clin Psychol. 2005;1: 255–291. 17716089 10.1146/annurev.clinpsy.1.102803.143948

[11] Agaibi CE, Wilson JP. Trauma, PTSD, and resilience: A review of the literature. Trauma, Violence, & Abuse. 2005;6: 195–216. 10.1177/1524838005277438 16237155

[12] Hoge EA, Austin ED, Pollack MH. Resilience: research evidence and conceptual considerations for posttraumatic stress disorder. Depress Anxiety. 2007;24: 139–152. 16892420 10.1002/da.20175

[13] Fredrickson BL. The broaden–and–build theory of positive emotions. Philosophical Transactions of the Royal Society of London B: Biological Sciences, 2004;359: 1367–1377. 15347528 10.1098/rstb.2004.1512 PMC1693418

[14] Hayes SC. Acceptance and commitment therapy, relational frame theory, and the third wave of behavioral and cognitive therapies. Behavior therapy. 2004;35: 639–665. 10.1016/j.beth.2016.11.006 27993338

[15] Diener E, Suh E, Lucas R, Smith H. Subjective well-being: Three decades of progress. Psychol Bull. 1999;125: 276–302.

[16] Kahneman D, Diener E, Schwarz N, editors. Well-Being: The Foundations of Hedonic Psychology New York: Rusell Sage Foundation; 2003.

[17] United Nations Development Programme. Human Development Report 2015 New York: PBM Graphics; 2015.

[18] CBS. Welzijn in Nederland ("Wellbeing in the Netherlands"). Den Haag, Netherlands: Centraal Bureau voor de Statistiek (CBS); 2015.

[19] Office for National Statistics. Personal Well-being in the UK, 2014/15. Statistical Bulletin, September 23, 2015. http://www.ons.gov.uk/ons/dcp171778_417216.pdf

[20] van Dierendonck D. van Dierendonck D. The construct validity of Ryff's scales of psychological well-being and its extension with spiritual wellbeing. Pers Ind Diff. 2005;36:629–643.

[21] Krieke LVD, Jeronimus BF, Blaauw FJ, Wanders RBK, Emerencia AC, Schenk HM, et al HowNutsAreTheDutch (HoeGekIsNL): A crowdsourcing study of mental symptoms and strengths. Int J Meth Psychiat Res. 2015, in press. 10.1002/mpr.1495 PMC6877205 26395198

[22] Priebe S, Huxley P, Knight S, Evans S. Application and results of the Manchester Short Assessment of Quality of Life (MANSA). Int J Soc Psychiatry. 1999;45: 7–12. 10443245 10.1177/002076409904500102

[23] Abdel-Khalek A. Measuring happiness with a single-item scale. Soc Beh Pers. 2006;34: 139–149.

[24] Nieboer A, Lindenberg S, Boomsma A, Van Bruggen A. Dimensions of well-being and their measurement: The SPF-IL Scale. Soc Indicators Res. 2005;73: 313–353.

[25] Fordyce MW. A Review of Research on the Happiness Measures: A Sixty Second Index of Happiness and Mental Health. Soc Indicators Res. 1988;20: 355–381.

[26] Oishi S, Diener E, Lucas RE. The Optimum Level of Well-Being: Can People Be Too Happy? Persp Psychol Sci. 2007;2: 346–360. 10.1111/j.1745-6916.2007.00048.x 26151972

[27] Nettle D. Happiness: The Science Behind Your Smile New York: Oxford University Press; 2006.

[28] Diener E. Subjective well-being. Psychol Bull. 1984;95: 542–575. 6399758

[29] Lovibond SH, Lovibond PF. Manual for the Depression Anxiety Stress Scales. 2nd ed. Sydney: Psychology Foundation; 1995.

[30] De Beurs E, van Dyck R, Marquenie LA, Lange A, Blonk RWB. De DASS; een vragenlijst voor het meten van depressie, angst en stress. Gedragstherapie. 2001;34: 35–53.

[31] Rush A, Trivedi M, Ibrahim H, Carmody T, Arnow B, Klein D, et al The 16-item Quick Inventory of Depressive Symptomatology (QIDS), clinician rating (QIDS-C), and self-report (QIDS-SR): A psychometric evaluation in patients with chronic major depression. Biol Psychiatry. 2003;54: 573–583. 12946886 10.1016/s0006-3223(02)01866-8

[32] Peeters FPML, Ponds RHWM, Vermeeren MTG. Affectiviteit en zelfbeoordeling van depressie en angst. Tijdschrift voor de Psychiatrie. 1996;38.

[33] Clark LA, Watson D. Tripartite model of anxiety and depression—Psychometric evidence and taxonomic implications. J Abnorm Psychol. 1991;100: 316–336. 1918611 10.1037//0021-843x.100.3.316

[34] Crawford JR, Henry JD. The Depression Anxiety Stress Scales (DASS): Normative data and latent structure in a large non-clinical sample. Br J Clin Psychol. 2003;42: 111–131. 12828802 10.1348/014466503321903544

[35] Martin RA, Puhlik-Doris P, Larsen G, Gray J, Weir K. Individual differences in uses of humor and their relation to psychological well-being: Development of the Humor Styles Questionnaire. J Res Pers. 2003;37: 48–75.

[36] Baron-Cohen S, Wheelwright S. The Empathy Quotient: An investigation of adults with Asperger syndrome or high functioning Autism, and normal sex differences. J Autism Dev Disord. 2004;34: 163–175. 15162935 10.1023/b:jadd.0000022607.19833.00

[37] Peterson RA, Brown SP. On the use of beta coefficients in meta-analysis. J Appl Psychol. 2005;90: 175–181. 15641898 10.1037/0021-9010.90.1.175

[38] Richard FD, Bond CF Jr., Stokes-Zoota J. One hundred years of social psychology quantitatively described. Rev Gen Psychol. 2003;7: 331–363.

[39] Lovibond PF, Lovibond SH. The structure of negative emotional states—Comparison of the Depression Anxiety Stress Scales (Dass) with the Beck Depression and Anxiety Inventories. Behav Res Ther. 1995;33: 335–343. 7726811 10.1016/0005-7967(94)00075-u

[40] Bergsma A, Veenhoven R, ten Have M, de Graaf R. Do they know how happy they are? On the value of self-rated happiness of people with a mental disorder. J Happiness Stud. 2011;12: 793–806.

[41] American Psychiatric Association. Diagnostic and Statistical Manual of Mental Disorders. 5th ed: Washington DC, Author; 2013.

[42] Kapur S, Phillips AG, Insel TR. Why has it taken so long for biological psychiatry to develop clinical tests and what to do about it? Mol Psychiatry. 2012;17: 1174–1179. 10.1038/mp.2012.105 22869033

[43] Kendler KS, Zachar P, Craver C. What kinds of things are psychiatric disorders? Psychol Med. 2011;41: 1143–1150. 10.1017/S0033291710001844 20860872

[44] Lewinsohn PM, Sullivan JM, Grosscup SJ. Changing reinforcing events: An approach to the treatment of depression. Psychotherapy: Theory, Research and Practice. 1980;17: 322–334.

[45] McConnell AR, Brown CM, Shoda TM, Stayton LE, Martin CE. Friends with benefits: On the positive consequences of pet ownership. J Pers Soc Psychol. 2011;6: 1239–1252. 10.1037/a0024506 21728449

[46] Allen K, Blascovich J. The value of service dogs for people with severe ambulatory disabilities—A randomized controlled trial. JAMA. 1996;275: 1001–1006. 8596231

[47] Catalino LI, Algoe SB, Fredrickson BL. Prioritizing positivity: An effective approach to pursuing happiness? Emotion. 2014;14: 1155–1161. 10.1037/a0038029 25401290 PMC5533095

[48] Burrows KE, Adams CL, Spiers J. Sentinels of safety: Service dogs ensure safety and enhance freedom and well-being for families with autistic children. Qual Health Res. 2008;18: 1642–1649. 10.1177/1049732308327088 18955467

[49] Taylor SE. Tend and Befriend: Biobehavioral bases of affiliation under stress. Current Directions in Psychol Sci. 2006;15: 273–277.

[50] Snippe E, Jeronimus BF aan het Rot M, Bos EH, de Jonge P, Wichers M. Prosocial people smile: the daily dynamics of an upward spiral. Submitted.

[51] Henman LD. Humor as a coping mechanism: Lessons from POWs. Int J Humor Res. 2008;14: 83–94.

[52] Connor K, Davidson JT, Lee L. Spirituality, resilience, and anger in survivors of violent trauma: A community survey. J Trauma Stress. 2003;16: 487–494. 14584633 10.1023/A:1025762512279

[53] Heintzelman SJ, Trent J, King LA. Encounters with objective coherence and the experience of meaning in life. Psychol Sci. 2013;24: 991 10.1177/0956797612465878 23620548

[54] Jeronimus BF, Riese H, Sanderman R, Ormel J. Mutual reinforcement between neuroticism and life stressors: A five-wave, sixteen-year study to test reciprocal causation. JPSP. 2014;107: 751. 10.1037/a0037009 25111305

[55] Krueger RF, Markon KE. Reinterpreting comorbidity: A model-based approach to understanding and classifying psychopathology. Annu Rev Clin Psychol. 2006;2: 111 17716066 10.1146/annurev.clinpsy.2.022305.095213 PMC2242354

[56] Kendler KS, Parnas J, editors. Philosophical Issues in Psychiatry III: The Nature and Sources of Historical Change United Kingdom: Oxford University Press; 2015.

[57] Salloum IM, Mezzich JE. Outlining the bases of person-centred integrative diagnosis. J Eval Clin Pract. 2011;17: 354–356. 10.1111/j.1365-2753.2010.01581.x 21114718

[58] IGDA Workgroup W. IGDA. Essentials of the World Psychiatric Association's International Guidelines for Diagnostic Assessment. Brit J Psychiat. 2003;182: s37–s66.

[59] Bolier L, Haverman M, Westerhof G, Riper H, Smit F, Bohlmeijer E. Positive psychology interventions: a meta-analysis of randomized controlled studies. BMC Public Health. 2013;13: 1–20. 10.1186/1471-2458-13-119 23390882 PMC3599475

[60] Mead N, Lester H, Chew-Graham C, Gask L, Bower P. Effects of befriending on depressive symptoms and distress: systematic review and meta-analysis. Brit J Psychiat. 2010;196: 96–101. 10.1192/bjp.bp.109.064089 20118451

[61] Sin NL, Lyubomirsky S. Enhancing well-being and alleviating depressive symptoms with positive psychology interventions: a practice-friendly meta-analysis. J Clin Psychol. 2009;65: 467–487. 10.1002/jclp.20593 19301241

[62] Smout MF, Hayes L, Atkins PWB, Klausen J, Duguid JE. The empirically supported status of acceptance and commitment therapy: An update. Clinical Psychologist. 2012;16: 97–109.

[63] Lyubomirsky S, Sheldon K, Schkade D. Pursuing happiness: The architecture of sustainable change. Rev Gen Psychol. 2005;9: 111–131.

[64] Rafaeli E, Rogers GM, Revelle W. Affective Synchrony: Individual Differences in Mixed Emotions. Pers Soc Psychol Bull. 2007;33: 915–932. 17551163 10.1177/0146167207301009

