# Supplementary material for: Preserving Subjective Wellbeing in the Face of Psychopathology: Buffering Effects of Personal Strengths and Resources
Source: PLoS One. 2016 Mar 10;11(3):e0150867. doi: 10.1371/journal.pone.0150867 (PMC4786317; doi:10.1371/journal.pone.0150867)
Supplement: S1 Text — (DOC) [file pone.0150867.s001.doc]

**Module Leefomstandigheden en tijdsbesteding (minimum 19, maximum 26 vragen)**

U heeft al wat vragen beantwoord over uw demografie. De volgende vragen gaan hier wat dieper op in en gaan over uw afkomst, leefomstandigheden, vrijetijdsbesteding en uw fysiek.

**9. Wat is uw oorspronkelijke land of gebied van herkomst?**

- Nederland
- Anders

|  | - - Ander Europees land dan Nederland   - Marokko   - Turkije   - Suriname   - (voormalig) Nederlandse Antillen /Aruba   - Noord-Afrika   - Zuid-Afrika   - Azië   - Latijns-Amerika   - Noord-Amerika/Australië/Nieuw-Zeeland   - Onbekend |
| --- | --- |

**10. Wat is het oorspronkelijke land of gebied van herkomst van uw vader?**

- Nederland
- Anders

|  | - - Ander Europees land dan Nederland   - Marokko   - Turkije   - Suriname   - (voormalig) Nederlandse Antillen /Aruba   - Noord-Afrika   - Zuid-Afrika   - Azië   - Latijns-Amerika   - Noord-Amerika/Australië/Nieuw-Zeeland   - Onbekend |
| --- | --- |

**11. Wat is het oorspronkelijke land of gebied van herkomst van uw moeder?**

- Nederland
- Anders

|  | - - Ander Europees land dan Nederland   - Marokko   - Turkije   - Suriname   - (voormalig) Nederlandse Antillen /Aruba   - Noord-Afrika   - Zuid-Afrika   - Azië   - Latijns-Amerika   - Noord-Amerika/Australië/Nieuw-Zeeland   - Onbekend |
| --- | --- |

**12. Waaruit bestaat uw huidige huisvesting?**

- Koopwoning
- Huurwoning
- Ouderlijk huis (inwonend bij ouders)
- Op kamers
- Beschermd wonen project
- Niet-GGZ-institutie (internaat, gevangenis, AZC)
- GGZ-institutie
- Pension / kosthuis
- Zwervend / dakloos /tehuis voor dak- en thuislozen / sociaal pension
- Woongroep
- Anders

**13. Wat is het netto maandinkomen van uw huishouden?**

(Netto is het bedrag dat u maandelijks op uw rekening krijgt. Als u het huishouden met iemand deelt, dan ook de inkomsten van uw partner meetellen.)

- minder dan € 750
- € 751 - € 1000
- € 1001 - € 1500
- € 1501 - € 2000
- € 2001 - € 2500
- € 2501 - € 3000
- € 3001 - € 3500
- Meer dan € 3500
- Ik weet het niet
- Wil ik liever niet zeggen

**14. Uit hoeveel mensen bestaat uw huishouden (uzelf meegerekend)?**

|  |  | mensen |
| --- | --- | --- |

**15. Met welke personen woont u samen? (meerdere antwoorden mogelijk)**

- met een partner
- met kind(eren)
- met mijn ouder(s)
- met andere volwassenen die niet mijn ouders of partner zijn
- ik woon alleen

**16. Heeft u huisdieren?**

- Ja

|  | **Wat voor huisdier(en) heeft u?**   - - Hond(en)   - Kat(ten)   - Vogels   - Knaagdieren (Cavia, konijn, muizen, ratten)   - Reptielen   - Vissen   - Anders |
| --- | --- |

- nee

**17. Hoeveel kinderen zijn er geboren in het gezin waaruit u komt, uzelf meegerekend?**

|  |  | kinderen |
| --- | --- | --- |

**18. Als hoeveelste kind bent u zelf geboren?**

|  |  |
| --- | --- |

**19. Bent u deel van een meerling?**

- ja
- nee
- weet ik niet

**20. Behoort u tot een kerkelijke of levensbeschouwelijke stroming/gemeenschap?**

- Protestant-christelijk

|  | **Gaat u wel eens naar een samenkomst van de voorgenoemde gemeenschap? En zo ja, hoe vaak?**   - nooit - jaarlijks of minder vaak - een aantal keer per jaar - maandelijks - 2-3 keer per maand - wekelijks of vaker |
| --- | --- |

- Rooms-katholiek

|  | **Gaat u wel eens naar een samenkomst van de voorgenoemde gemeenschap? En zo ja, hoe vaak?**   - nooit - jaarlijks of minder vaak - een aantal keer per jaar - maandelijks - 2-3 keer per maand - wekelijks of vaker |
| --- | --- |

- Jodendom

|  | **Gaat u wel eens naar een samenkomst van de voorgenoemde gemeenschap? En zo ja, hoe vaak?**   - nooit - jaarlijks of minder vaak - een aantal keer per jaar - maandelijks - 2-3 keer per maand - wekelijks of vaker |
| --- | --- |

- Islam

|  | **Gaat u wel eens naar een samenkomst van de voorgenoemde gemeenschap? En zo ja, hoe vaak?**   - nooit - jaarlijks of minder vaak - een aantal keer per jaar - maandelijks - 2-3 keer per maand - wekelijks of vaker |
| --- | --- |

- Hindoeïsme

|  | **Gaat u wel eens naar een samenkomst van de voorgenoemde gemeenschap? En zo ja, hoe vaak?**   - nooit - jaarlijks of minder vaak - een aantal keer per jaar - maandelijks - 2-3 keer per maand - wekelijks of vaker |
| --- | --- |

- Boeddhisme

|  | **Gaat u wel eens naar een samenkomst van de voorgenoemde gemeenschap? En zo ja, hoe vaak?**   - nooit - jaarlijks of minder vaak - een aantal keer per jaar - maandelijks - 2-3 keer per maand - wekelijks of vaker |
| --- | --- |

- Humanisme

|  | **Gaat u wel eens naar een samenkomst van de voorgenoemde gemeenschap? En zo ja, hoe vaak?**   - nooit - jaarlijks of minder vaak - een aantal keer per jaar - maandelijks - 2-3 keer per maand - wekelijks of vaker |
| --- | --- |

- Anders

|  | **Gaat u wel eens naar een samenkomst van de voorgenoemde gemeenschap? En zo ja, hoe vaak?**   - nooit - jaarlijks of minder vaak - een aantal keer per jaar - maandelijks - 2-3 keer per maand - wekelijks of vaker |
| --- | --- |

- Geen

**21. Hoeveel van uw vrije tijd besteedt u gemiddeld per dag aan televisie, tablet en computer?**

|  |  | uur en |  |  | minuten |
| --- | --- | --- | --- | --- | --- |

**22. Hoeveel tijd sport u gemiddeld per week?**

|  |  | uur en |  |  | minuten |
| --- | --- | --- | --- | --- | --- |

**23. Hoeveel kilo weegt u (zonder kleren, in hele kilogrammen)?**

|  |  |  |
| --- | --- | --- |

**24. Hoe lang bent u (zonder schoenen, in centimeters)?**

|  |  |  |
| --- | --- | --- |

**25. Bent u links- of rechtshandig?**

- linkshandig
- rechtshandig
- zowel links- als rechtshandig
